# Optical Coherence Tomography Position Analysis of Retropupillary Iris-Fixated Intraocular Lens in Iris Tissue

**DOI:** 10.1155/2022/5948208

**Published:** 2022-08-09

**Authors:** Jana C. Riedl, Michael R. Bopp, Charlotte F. Gross, Urs Vossmerbaeumer

**Affiliations:** Department of Ophthalmology, University Medical Center, Johannes Gutenberg-University Mainz, Mainz, Germany

## Abstract

**Purpose:**

The aim of this study was to evaluate a micrometer-accurate analysis of the retropupillary Verisyse® intraocular lens (IOL) (Ophtec, Groningen NL; Santa Ana, USA) enclavation in the iris tissue.

**Methods:**

A retrospective consecutive case series was conducted at the Department of Ophthalmology, University Hospital Mainz. Patients with an optical coherence tomography (Spectralis®, Heidelberg Engineering®; Heidelberg, Germany) examination after retropupillary Verisyse® IOL implantation were included retrospectively. The enclavation geometry was measured using the Eye-Explorer® from Heidelberg Engineering® (Heidelberg, Germany). Seventeen measuring positions were determined nasally and temporally at the corresponding enclavation points.

**Results:**

72 eyes of 67 patients after implantation of a retropupillary Verisyse® IOL were analyzed. The average age was 68 ± 17.2 years (63% female; 38% male). The analysis of the position of the Verisyse*®* IOL showed highly homogeneous data in all measured points. The depth of the anterior chamber showed a positive correlation with width of the posterior deflection of the iris tissue behind the enclavation (Pearson *r*: 0.321, *p*=0.041). The offset of the haptics showed greater deviations, and the lens diopter implanted was higher (*r* = 0.337, *p*=0.007).

**Conclusion:**

This is the first study that analysis the exact enclavation of retropupillary implanted Verisyse® IOL. It provides new information about the intrastromal course of the haptics in the iris tissue. It could be shown that the haptics do not run parallel in the iris tissue, but are anchored in the iris tissue with an average offset of 95 *µ*m. This rebuts previous assumptions about the intrastromal course and provides new information.

## 1. Introduction

The retropupillary implantation of an iris-fixated intraocular lens (IOL) is a minimally invasive standard procedure as a primary or secondary operation to correct aphakia. In 1971, the lens model has been implanted retropupillary inverse for the first time, but it took until 2002 to established this technique for optical rehabilitation when the capsular apparatus was missing [1, 2]. Over the last years, different techniques for aphakia correction have been developed. The initial situation (including the degree of capsular bag and iris defects) must be taken into account for choosing the type of correction. Anterior chamber IOL has the advantage of small learning curve, faster surgical time, and less complications like vitreous hemorrhage and suture erosion compared to scleral-fixated IOLs [3, 4]. In cases of both, capsular and iris deficiency, glued IOL with aniridia IOL, or iridoplasty with glued IOLs showed good functional and anatomical results [5]. With a low intraoperative and postoperative risk profile, the iris-claw lens replaced the normally applied scleral-suture-fixed implantation of a posterior chamber lens [2, 6]. Furthermore, the time of operation and the learning curve for new surgeons are short [6]. An intact iris structure has prerequisites, at least at the enclavation positions [7]. The surgical method is already described in detail and special instruments, such as a specially developed forceps and spatula for easy retropupillary implantation of iris-claw lenses in aphakia (Geuder GmbH and Heidelberg) have been developed [7, 8]. A limited refractive predictability caused by an individual variable of the anterior chamber due to retracting iris tissue and head position should always be mentioned before the operation [2, 9]. Furthermore, the mechanical stability is dependent on the amount of enclavated iris tissue and the vitreous body status [10]. To improve the target refraction, several working groups developed adapted A-constants [7, 10]. There are also lots of studies evaluating the efficiency and safety of iris-claw lenses [11–13]. However, none of the studies deal with the exact enclavation of the IOL in the iris tissue. Typically, the enclavation is evaluated indirectly using a slit lamp. Using spectral domain optical coherence tomography (SD-OCT, Spectralis®OCT, Heidelberg Engineering), a precise evaluation should be carried out with regard to the assessment of the enclavation of the haptics on the posterior side of the iris and in the iris tissue. So, new information can be obtained about the intrastromal course and the point of contact of the branches in the iris pigment epithelium and stroma.

## 2. Methods

This retrospective case series was performed at the Department of Ophthalmology, University Medical Center of the Johannes Gutenberg University Mainz, where all patients were evaluated. The study was conducted according to the tenets of the Declaration of Helsinki. The inclusion criteria were as follows: male and female; having had a mono- or bilateral retropupillary iris-fixated IOL implantation; OCT examination of the iris enclavation two days postoperative. Exclusion criterion were as follows: no visualization of the IOL enclavation postoperative.

**Figure 1:**
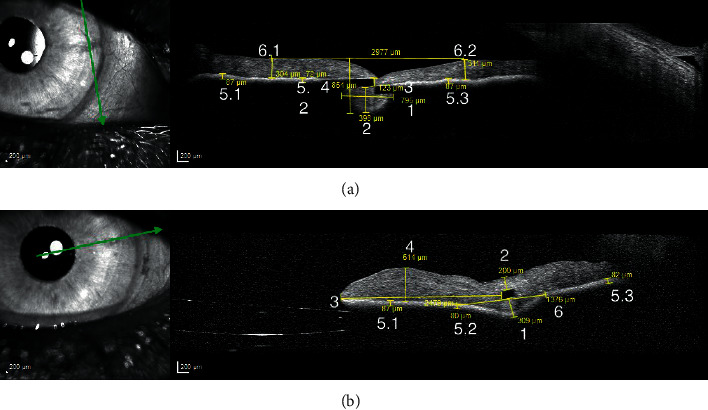
(a) Nine measuring points on the longitudinal axis shown for a haptic in the 3'o clock position. (b) Eight measuring points on the transversal axis shown for a haptic in the 3'o clock position.

All patients were exanimated with the anterior segment mode of a SD-OCT (Spectralis, Heidelberg Engineering, Heidelberg, Germany) two days postoperative after retro-iridial implantation of a Verisyse® IOL. Using the measurement-tools of the Heidelberg Eye Explorer, both enclavation positions, 3 and 9'o clock, in longitudinal and transversal directions were analyzed, Figure 1. The measuring points were defined as followed. Furthermore, patient's history was evaluated to analysis previous ocular disease.

### 2.1. Longitudinal Axis

1. Width of the iris tissue behind the enclavation
2. Depth of the iris tissue behind the enclavation
3. Offset of the haptic
4. Distance tangent of the highest neighboring points on the iris to the lowest point of the enclavated iris tissue (right-angled)
5. Thickness of the iris pigment epithelium on both sides of the IOL enclavation at three different points (5.1, 5.2, and 5.3)
6. Iris tissue of the thickest position on both sides of the enclavation

### 2.2. Transversal axis

1. Iris stroma behind the IOL-haptic
2. Iris stroma in front of the IOL-haptic
3. Distance between the inner haptic boundary and the pupillary zone
4. Thickest iris tissue between IOL-haptic and the pupillary zone
5. Thickness of the iris pigment epithelium on both sides of the IOL enclavation at three different points (5.1, 5.2, and 5.3)
6. Width of the posterior deflection of the iris tissue behind the enclavation

### 2.3. Surgery

A detailed description of the surgery is described in [7, 14].

### 2.4. SD-OCT

Noncontact imaging of the anterior eye was performed with a SD-OCT. SD-OCT provides enhanced visualization of anterior chamber structures, from the anterior surface of the cornea to the posterior surface of the lens. A detailed visualization of the iris helps to evaluate the surgical result after iris-fixated IOLs.

### 2.5. Statistical Methods

For descriptive analyses, mean values and standard deviations (SD) were calculated for all patients.

The SPSS program (IBM Corp., 2012, IBM SPSS Statistics for Windows, version 26.0, Armonk, NY) was used for statistical analysis.

The correlation coefficient according to Pearson and the graphic of a point-scatter diagram are selected for the representation of the relationship for continuous variables.

## 3. Results

**Figure 2:**
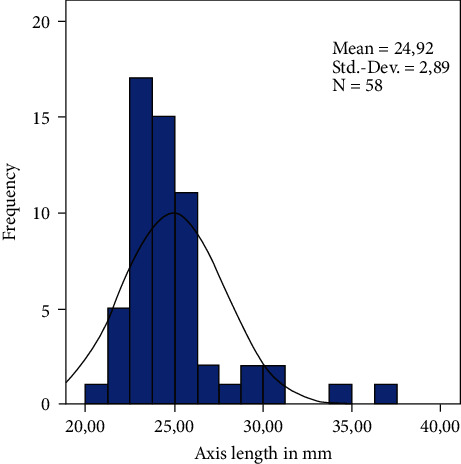
Distribution of the axial length in mm.

**Figure 3:**
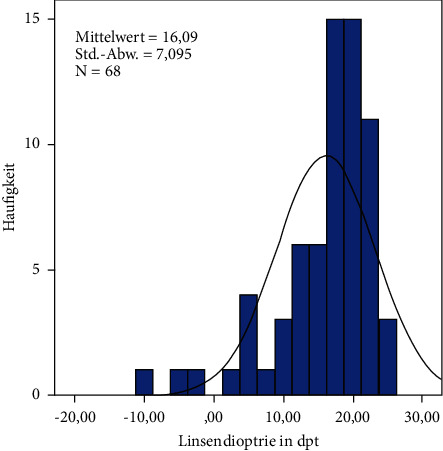
Distribution of the implanted lens (dioptres).

**Figure 4:**
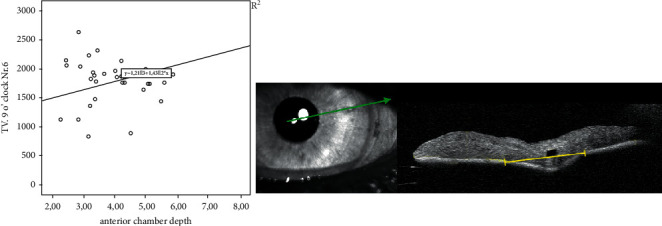
Relationship between anterior chamber depth and width of the posterior deflection of the iris tissue behind the IOL haptic shown with Pearson's correlation and the corresponding OCT picture of the measuring point (transversal).

**Figure 5:**
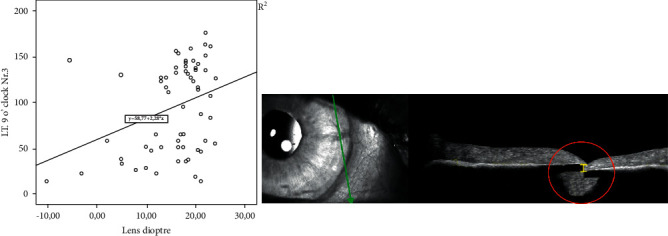
Relationship between the IOL diopter and width offset of the IOL haptic shown with Pearson's correlation and the corresponding OCT picture of the measuring point (longitudinal).

**Figure 6:**
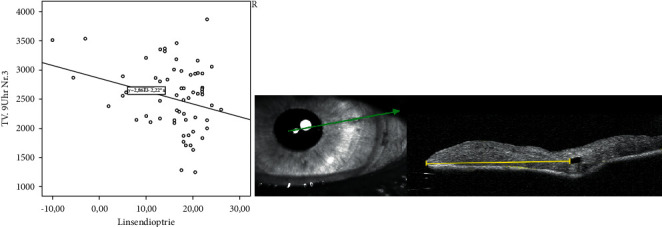
Relationship between IOL diopter and the distance between the inner haptic boundary and the pupillary zone (transversal).

In this study, we analyzed 72 eyes of 67 patients after retropupillary Verisyse® IOL implantation. 42 patients (63%) were male and 25 (38%) were female with an average age of 68 years ±17.2 (range: 12–90 years). 18 (25%) patients had PEX-syndrome, 22 (30%) had a prior vitrectomy, and 12 patients (17%) had a prior ocular trauma. The mean axis length of all eyes was 25 mm ± 2.89 (range: 21–37.8 mm), Table 1 and Figure 2, with a mean anterior chamber depth of 4.1 mm (range: 2.26–7.05 mm). The average diopter of implanted Verisyse®IOLs was +16D ± 7 (range −10D-+26D), as shown in Figure 3. All measured points (longitudinal and transversal) are shown in Table 2 for the 9'o clock position and 3'o clock position. The axial length showed no significant correlation with the measured points. The depth of the anterior chamber showed a positive correlation with width of the posterior deflection of the iris tissue behind the enclavation (Pearson *r*: 0.321, *p*=0.041), as shown in Figure 4. The greater the deviations of offset of the haptics, the higher the lens diopter implanted (*r* = 0.337, *p*=0.007), as shown in Figure 5. Furthermore, the higher the implanted lens diopter, the shorter the distance between the inner haptic boundary and the pupillary zone (*r* = −0.274, *p*=0.026), as shown in Figure 6. There was no correlation between eyes with a previous vitrectomy and the measured points. Eyes with a previous trauma had thicker iris tissue next to the enclavation (*r* = 0.337, *p*=0.0006).

## 4. Discussion

There are several new findings in the present study. First of all, the analysis of the position of the Verisyse® IOL measured with OCT showed highly homogeneous data in all measured points. Second, we could show that the deeper the anterior chamber, the broader the iris tissue behind the enclavation. A positive correlation was proved with the Pearson's correlation coefficient (r: 0.321, *p*=0.041). Third, there is an offset of the two branches of the haptics after enclavation in the iris tissue. The data of the present study support our hypothesis that the enclavation of a retropupillary IOL is a standardizes method for all surgeons. This is the first study that investigate the exact position of the Verisyse® IOL in the iris tissue. Most of the previous studies about retropupillary enclavated IOL's examined the refractive outcome, and compared the surgery technique with other aphakia corrections and dealt with the visual outcomes and complications [15–17]. Just very few studies analyzed the position of the IOL. Schoepfer et al. described the position-dependent accommodative sift of the retropupillary fixated iris-claw lens with a significant difference in the anterior chamber depth for backward- and forward-tilted heads and a resulting change in refraction [9]. There are also descriptions of a deeper anterior camber and a lager distance of the haptics from the corneal endothelium and a larger angle opening distance to the posterior plane of the iris in eyes with Verisyse® IOL enclavation than in eyes with anterior enclavation of the IOL measured with ultrasound biomicroscopy [18]. Baykara et al. and Rastogi et al. used pentacam and ultrasound biomicroscopy to show a centered IOL, which is also parallel to the iris plane with no tilt seen in all patients [12, 19]. There is one study using anterior segment OCT to evaluate the intraocular architecture of secondary implanted iris-claw lenses in aphakic eyes, but the IOLs were implanted in the anterior chamber and the measurement also just focused on the anterior chamber [20]. According to the authors, the comparison between smaller (axial-length <24 mm) and longer eyes in regard to the postoperative anterior chamber depth was significant different in their study, which might indicate posterior enclavation in smaller eyes [18].

With our measurements and data, we could show that the implantation of the retropupillary Verisyse® IOL is a highly standardized procedure with minor deviations independent of the surgeon and enclavation position (3'o clock vs. 9 o'clock). The standardized procedure result is rare intraoperative and postoperative complications and is comparable to standard cataract surgery [15]. Enclavation problems, hyphema, insufficient woundclosure, and vitreous hemorrhage can occur [17]]. Light pupil ovalisation is reversible during the first postoperative year, and iris pigment atrophies of the enclavation site had no clinical significance [15]. Even with significant previous damage to the eye such as PEX, trauma, or even after vitrectomy, sufficient enclavation and a stable fit can still be guaranteed in all cases.

A positive correlation was found between the depth of the anterior chamber and the width of the posterior deflection of the iris tissue behind the IOL-haptic, which means the deeper the anterior chamber the more iris tissues are enclavated. This could be explained with the anatomical conditions. If the anterior chamber is deeper, the instruments will likely be brought steeper into the eye. Therefore, more iris tissues are gripped and enclavated compared to eyes with a flatter anterior chamber.

Of note, there is an offset of the two claws after enclavation in the iris tissue. The higher the implanted lens diopter, the greater the offset of the two claws. This is of great interest for us. So far, we have assumed that the two claws run almost parallel in the iris tissue. There are neither studies for the position in the iris tissue of anterior iris-fixated lens nor retropupillary fixated lens, so no comparison with other data can be made. Yu et al. used Scheimpflug photography and ultrasound biomicroscopy to evaluate the position of the myopic iris-claw phakic intraocular lens but focusing of the anterior chamber, the distance to the corneal endothelium, and the distance between the superior and inferior optic edge and iris [21]. No statements were made of the intrastromal course. The offset of the two claws could be due to the construction of the Verisyse® itself. According to the manufacturer, the distance between the optical back and the end of the haptic depends on the chosen diopter. This results in a curved shape of the IOL (the more the diopter, the more curved the shape is), which could have an influence on the enclavation. The high torsional stiffness of the haptics, which, like the entire lens, consists of rigid polymethyl methacrylate (PMMA), which could be the reason of the increasing displacement of the haptics with increasing diopter. Weather this has a clinical influence, for example, an increased risk of subluxation, cannot be determined from this study. Gonnermann et al. reported disenclavation in 12 out of 137 patients (8.7%), which occurred at 3.3 month after surgery, so follow-up studies would be of great interest weather there are any connections between Verisyse® IOL enclavation position and disenclavation [22].

In conclusion, to the best of our knowledge, this is the first study which describes the exact intrastomal position of retropupillary enclavated Verisyse® IOL. Here is new information about an intrastromal haptic offset, which was not known before. Altogether, the present study supports our hypothesis that the enclavation of retropupillary IOLs is a standardizes method for all surgeons.

## Figures and Tables

**Table 1:** Distribution of gender, age, axial length, and ocular diseases.

	*n*	%
Gender (f/m)	25/42	38/63
Age (years)	68 ± 17.2	
Pseudoexfoliation	18	25
Previous trauma	12	17
Previous vitrectomy	22	31
Axial length (mm)	24.9 ± 2.89	
Implanted Verisyse® (diopter)	16.1 ± 7.09	

**Table 2:** All measured points as described in the method sections for the longitudinal and transversal orientation for the 3'o clock position and 9'o clock position.

Axis	Measuring point	3'o clock Mean (*µ*m) ± SD min-max	9'o clock Mean (*µ*m) ± SD min-max
Longitudinal	1.	767 ± 321 (264–1810)	782 ± 236 (293–1535)
	2.	395 ± 124 (133–861)	403 ± 94 (160–626)
	3.	95 ± 45 (0–170)	96 ± 48 (14–176)
	4.	916 ± 130 (575–1333)	905 ± 130 (602–1239)
	5.1.	80 ± 15 (6–116)	82 ± 14 (47–130)
	5.2.	79 ± 13 (55–113)	82 ± 14 (51–114)
	5.3.	80 ± 13 (47–109)	83 ± 15 (53–137)
	6.1.	328 ± 65 (199–472)	316 ± 66 (182–440)
	6.2.	322 ± 64 (125–456)	327 ± 73 (271–318)

Transversal	1.	293 ± 91 (87–393)	320 ± 111 (105–607)
	2.	142 ± 61 (57–393)	135 ± 50 (52–260)
	3.	2502 ± 668 (1238–4346)	2497 ± 571 (1245–3869)
	4.	408 ± 79 (180–579)	408 ± 75 (211–605)
	5.1.	82 ± 13 (48–127)	81 ± 13 (48–120)
	5.2.	82 ± 14 (51–138)	81 ± 12 (50–125)
	5.3.	78 ± 11 (54–124)	81 ± 12 (58–109)
	6.1.	1683 ± 449 (0–2712)	1831 ± 414 (0–2630)

## Data Availability

The data used to support the findings of this study are available upon request.
